# Unequal Associations between Educational Attainment and Occupational Stress across Racial and Ethnic Groups

**DOI:** 10.3390/ijerph16193539

**Published:** 2019-09-21

**Authors:** Shervin Assari, Mohsen Bazargan

**Affiliations:** 1 Departments of Family Medicine, College of Medicine, Charles R Drew University of Medicine and Science, Los Angeles, CA 90059, USA; Mohsenbazargan@cdrewu.edu; 2 Departments of Family Medicine, University of California, Los Angeles (UCLA), Los Angeles, CA 90095, USA

**Keywords:** population groups, ethnicity, race, Hispanics, Latinos, Whites, Blacks, African Americans, socioeconomic position, socioeconomic status, education, stress, occupational stress, work, employment

## Abstract

Background: Although other mechanisms are also involved, at least one reason high educational attainment (EA) is associated with better health is lower employment stress in individuals with high EA. *Minorities’ Diminished Returns*, however, refer to the smaller protective health effects of EA for racial- and ethnic-minority individuals, particularly African Americans (AAs) and Hispanics, as compared to Whites. We are, however, not aware of many studies that have explored differential associations between EA and work-related stress across racial and ethnic groups. Aims: We aimed to compare racial and ethnic groups for the association between EA and occupational stress in a national sample of American adults. Methods: The National Health Interview Survey (NHIS 2015), a cross-sectional survey, included 15,726 employed adults. Educational attainment was the independent variable. Occupational stress was the outcome. Race and ethnicity were the moderators. Age, gender, number of jobs, and years in the job were the covariates. Results: Overall, higher EA was associated with lower levels of occupational stress. Race and ethnicity both interacted with EA, suggesting that the association between high EA and reduced occupational stress is systemically smaller for AAs and Hispanics than it is for Whites. Conclusions: In the United States, race and ethnicity limit the health gains that follow EA. While EA helps individuals avoid environmental risk factors, such as occupational stress, this is more valid for non-Hispanic Whites than AAs and Hispanics. The result is additional physical and mental health risks in highly educated AAs and Hispanics. The results are important, given racial and ethnic minorities are the largest growing section of the US population. We should not assume that EA is similarly protective across all racial and ethnic groups. In this context, EA may increase, rather than reduce, health disparities.

## 1. Introduction

According to the Minorities’ Diminished Returns (MDRs) theory [1,2,3,4,5], at least some of the racial/ethnic disparities are due to the “less than expected” protective effects of socioeconomic status (SES) indicators, such as educational attainment (EA) [1,6,7,8]. The MDRs theory suggests that (a) racial/ethnic health disparities are not all due to SES gaps but are also due to differential health gains that follow high SES for African American (AA) and Hispanic populations, (b) the racial and ethnic health inequalities tend to widen at higher SES levels, and (c) there is a need to address racial/ethnic disparities across all SES levels [1,2,3].

Some evidence suggests that MDRs hold for a wide range of health domains. Similar results are shown for a wide range of physical [9,10,11,12] and mental health [13,14] outcomes, meaning that high-SES AAs and Hispanics tend to have worse health when compared to high-SES Whites [13,15,16,17]. Very few studies, however, have explored the MDRs of EA on occupation characteristics. In one study, EA better reduced secondhand-smoke exposure at workplaces for Whites than at workplaces for AAs [18].

The US labor market is a dual system. One system is composed of low-stress, high-pay, prestigious jobs. Another system is composed of high-demand, high-stress, low-pay jobs. The US labor market is known to discriminate against racial and ethnic minorities and employ the majority of racial and ethnic minorities in the lower-tier jobs. That is, the labor market, similar to the broader society, tends to marginalize AAs and Hispanics [10,19]. A large body of research has shown that identical CVs and resumes generate very different outcomes for people, simply based on the racial and ethnic names [20]. As a result, highly educated AAs and Hispanics are less likely to secure high-quality occupations. As a result, highly educated AAs and Hispanics still work in jobs that are high in exposure to a wide range of stressors, toxicants, and undesired conditions [18,19]. One study was published recently, showing that highly educated AAs and Hispanics are more likely to be exposed to environmental exposures, such as secondhand smoke, than highly educated Whites [18]. In addition to the labor-market discrimination, residential segregation also plays a role. AAs and Hispanics are more likely to live in areas with poor job opportunities. Thus, highly educated AAs and Hispanics have a lower chance of accessing good occupations, even in the absence of labor-market discrimination [18,19]. Finally, the education is of lower quality for racial and ethnic minority groups. This may also result in differential association between educational attainment and occupation quality of highly educated AAs and Hispanics. All of these possibilities potentially explain the MDRs of educational attainment on health [1,6,7,18,21].

### Aims

In response to the gap in the literature on the contribution of occupational stress as an explanatory mechanism for MDRs, we conducted a study to explore race and ethnic variations in the association between EA and occupational stress in employed American adults. We had two hypotheses.

**Hypothesis** **1.** *We expected an inverse association between EA and occupational stress in the overall sample.*

**Hypothesis** **2.** *We expected the inverse association between EA and occupational stress to be smaller for AAs and Hispanics than for non-Hispanic Whites.*

## 2. Materials and Methods

### 2.1. Design and Settings

This is a secondary analysis of the 2015 National Health Interview Survey (NHIS) data, the main national health surveys of Americans, which are funded and conducted by the CDC [22]. The NHIS participants were eligible if they were (1) civilians, (2) non-institutionalized, (3) part of the US population, and (4) 18+ years of age. The NHIS sampling was a multistage, clustered, stratified probability sample. Although the NHIS 2015 includes 33,672 adults (from all races and ethnicities and including employed and non-employed), this analysis was limited to adults who were either White or AA and were employed. Thus, only 15,726 individuals entered our analysis.

### 2.2. Study Variables

The study variables included demographics (age and gender), race, ethnicity, EA (SES), region, years of employment, number of occupations, and occupational stress, which were all measured at an individual level. Race and ethnicity were self-identified and included AAs versus Whites and Hispanics versus non-Hispanics. Demographic Characteristics included age and gender. Age was a continuous measure, and gender was a dichotomous measure (male 1, female 0). Having more than one job was a dichotomous variable. Participants were asked if they were working at more than one job. Educational Attainment was a continuous measure, varying from 0 to 24 years. Marital Status was 1 = married versus 0 = any other status (never married, widowed, separated, divorced, and living with a partner). Region was operationalized as three binary variables: (1) the Northeast, (2) the Midwest, and (3) the South. The reference group was the West.

The outcome in this study was occupational stress, which was measured using the following items: (1) job interferes with personal or family life; (2) subject does not have enough time to get job done; (3) job does not allow for making decisions; (4) subject does not have support from supervisor when necessary; (5) workplace is unsafe; (6) employee health/safety is not important to management; (7) frequency of harassment at work; (8) frequency of job-required repeated activities, like lifting and pulling; and (9) frequency of job requiring standing or walking. Item responses ranged from 1 to 5, with 5 reflecting more stress. Our overall occupational stress score ranged from 1 to 5, which was average of all nine above items. (Cronbach alpha = 0.63)

This study measure was not a standardized measure but was conceptualized based on the following theoretical frameworks: minority stress theory, dual job market theory, job demands theory, and effort–reward imbalance. Minority stress theory explains that due to racism, discrimination, and prejudice, minority groups experience and perceive more stress than the mainstream populations, and the labor market is not an exception to this rule. Thus, AAs experience more harassment and discrimination in the workplace [23]. Based on the effort–reward imbalance, we can argue that non-Whites are rewarded less for the same amount of effort they put into their work. In this view, the economic and noneconomic return for occupational investment would be smaller for the members of the minority than for the majority groups [24]. That is, being a discriminatory system, the labor market tends to undervalue non-Whites’ contributions to the workforce. Job demands theory suggests that perceived occupational stress is high when the job is demanding and employees do not have access to the support that they need to successfully complete their occupational tasks. Such demands may be higher, and the provided support may be lower, in the occupations that AAs enter. In these jobs, the individuals perceive lower control over life and feel overburdened [25,26]. The dual job market theory argues that AAs and Hispanics enter second-tier jobs that are determined by a high workload, high stress, low benefits, and minimal pay. Thus, race and ethnicity impact occupational stress through types of occupations that each racial and ethnic group occupies [27,28,29].

### 2.3. Data Analytical Plan

We analyzed the data using SPSS 23.0 (IBM Corporation, Armonk, NY, USA), which enabled us to accommodate survey weights. First, we examined the distribution of our variables. Then, we used the Pearson correlation test to estimate univariate correlations between the study variables. To perform our multivariable analysis, linear regressions were used. Before running the models, we tested the assumptions and requirements, such as linear distribution of errors and no collinearity between independent variables. We ran models in the pooled sample, both with and without interaction terms between race and ethnicity with EA.

### 2.4. Ethics

According to the NIH and Charles R Drew University of Medicine and Science (CDU) definition of human subjects research, this study was found to be a non-human subject research, which is except from a Ethics Review Board (IRB) review. We used the NIH decision tool available here: https://grants.nih.gov/policy/humansubjects/research.htm. The NHIS main study protocol, however, was fully reviewed and approved by the Research Ethics Review Board (IRB) of the National Center for Health Statistics, CDC. All participants signed written informed consent. Participants were reminded that they had the right not to participate in the study, to cancel their participation at any moment, or to decline to answer any questions that they wished not to answer.

## 3. Results

### 3.1. Descriptive Statistics

Table 1 shows the descriptive statistics of our participants. This analysis included 15,726 American adults. This number was composed of Whites (13,322, 84.7%) or AAs (2398, 15.3%). This sample was mostly non-Hispanic (13,741, 87.6%) and only 1951 individuals (12.4%) were Hispanic. The mean age of our participants was 43.4 (SD = 14.2) years. The mean EA of the participants was 15.8 years (SD = 2.8 years).

### 3.2. Bivariate Analysis

Table 2 shows zero order (unadjusted) correlations between the study variables. We found positive correlations between race (AAs) and ethnicity (Hispanics) with occupational stress. Age, EA, and income were negatively correlated with occupational stress (Table 2).

### 3.3. Multivariable Analysis

Table 3 shows the summary of the results of two linear regression models, with EA as the independent variable and occupational stress as the dependent variable. These models were run in the overall sample. Model 1 focused on the main effects of EA, race, ethnicity, and covariates. Model 2 had those variables in addition to interaction terms between race and ethnicity with EA. Based on Model 1, high EA reduced occupational stress; however, based on Model 2, this association was weaker for AAs and Hispanics than it was for Whites and non-Hispanics (Table 3).

## 4. Discussion

Two main findings were observed. First, in general, a high EA was associated with less occupational stress. Second, this association was a function of race and ethnicity. The smaller inverse association between a high EA and reduced occupational stress in non-Whites offers a potential explanation for the MDRs of educational attainment, employment, and even income on the health of AAs and Hispanics.

The results of this study can also be understood as being in line with other empirical studies in the field [30,31]. In such studies, the main findings demonstrate consistent associations between higher education and lower levels of work stress in all countries. The strength of this association, however, varies across countries and is comparatively small in countries offering pronounced ‘integrative’ policies, in terms of high investments into measures of an active labor market policy and high participation rates in lifelong learning activities [32]. The results pointed to different types of policies that may help to reduce educational differences in work stress, in particular policies supporting those who are disadvantaged in the labor market. Overall political climate [33,34,35,36] and welfare state [37,38,39] also impact occupational stress across racial and ethnic groups.

The results are important because national and local policy regulations can be implemented to minimize the MDRs of EA on occupational stress for AAs and Hispanics. This is because occupational stress can be manipulated by policies such as affirmative action, minimum pay, and benefits associated with jobs. The elimination of discrimination in the labor market can equalize the stressful conditions of occupations for racial groups so that we can expect Blacks, Hispanics, and Whites to experience the same level of occupational stress at each socioeconomic level. These policy changes may reduce inequalities that are due to the stressful work of AAs and Hispanics.

The results are similar to another recent study that EA better reduced any and daily secondhand-smoke exposure at work for Whites than it did for AAs and Hispanics [18]. Thus, occupational and employment conditions may be one of the mechanisms in which EA shows smaller health effects for AAs and Hispanics than for Whites, as shown by several previous studies [1,7,8,15,18,21,40]. The results may also explain why employment gives a greater life expectancy to White men and women and almost no additional years of life expectancy to AA men. In another study, for AAs, an increase in EA meant a higher likelihood of working in a White workplace and having more White coworkers. Such an increase in exposure to Whites in the workplace was associated with an increase in received discrimination. We can now understand why EA [1,7,8,15,18,21,40], generated income [9,14,41], and employment [10,42] bring vastly different health effects to Whites and AAs.

The MDRs literature has shown that EA and income have weaker effects on reducing the risk of obesity, chronic disease, depression, self-rated health, and mortality for AAs compared to Whites [13,15,43,44]. That is, highly educated AAs are more obese [9], more depressed [14,45], have more CMCs [46], report worse health [19,47], and die earlier [48] when compared to highly educated Whites. The current results suggest that at least some of the MDRs are because highly educated AAs have higher occupational stress than whites. That is, highly educated AAs enter jobs that are associated with higher levels of stress.

EA does not lower occupational stress for AAs. That means that highly educated AAs still have high levels of job stress. As a result, high-SES AAs and Hispanics, as described by the MDRs theory [1,2,3], would show a worse-than-expected health status [1,7,8,15,18,21,40]. Obesity [12], depression [14], anxiety [49], self-rated health [47], and chronic disease [46] are all worse than expected in highly educated AAs than in Whites. Similar patterns for various health outcomes suggest that the processes that cause MDRs are upstream and impact almost all health outcomes. At least some of the processes that cause racial and ethnic health disparities happen in the society, rather than the health-care system [50,51,52,53,54].

High occupational stress of highly educated AAs may be due to multiple factors. Given the existing residential segregation, highly educated AAs do not have access to high-paying, low-stress jobs. In addition to job segregation, due to labor-market discrimination, highly educated AAs and Hispanics enter the types of jobs that pay less and have more stress. Residential segregation also reduces the quality of education in the AA and Hispanic communities. As the quality of education is a determinant of the quality of occupation, AAs enter worse jobs than Whites.

As a result, there is a need to reduce MDRs through multiple policies. The solution should not be limited to equalizing SES, such as EA and employment, but also qualitatively reducing the gap between the education and employment of racial and ethnic groups. Higher enforcement of antidiscrimination laws should apply to the sectors of the society that are involved in EA and employment. At the same time, AA individuals should be helped to secure higher-paying, lower-stress jobs. Such solutions to MDRs may be needed to eliminate health disparities.

### 4.1. Implications

The results may help create policies and programs that improve structural and institutional barriers in the lives of AAs and Hispanics. There is a need for bold, innovative social and economic policies. Policy makers may run an “impact analysis” of their employment and labor policies, not just on the overall population but also on how such policies help generate or close the racial and ethnic gaps in health. Enforcing the existing antidiscrimination laws and penalizing the section of the labor market that does not adhere to antidiscrimination laws may equalize employment of racial and ethnic groups. Policies and programs are also needed to help AAs and Hispanics secure better jobs.

### 4.2. Future Research

There is a need to conduct more research on the solutions to occupational stress in AA and Latino communities. We also do not know what health problems, both mental and physical, are shaped by the additional occupational stress of the AA community. We also do not know how we can equalize race/ethnicity in terms of occupational stress.

It is also necessary to verify these results through qualitative research conducted on a small representative sample of minority groups. Thus, further investigation of the causes of these results, using various methods, is needed. There is also a need to study the implications that the results of this research will have on policy makers in the labor market.

### 4.3. Limitations

This study had some methodological limitations. The cross-sectional design of our data does not allow for causal inferences. The outcome was measured using single-item measure. We only measured workplace secondhand exposure. The sample size was imbalanced across race and ethnic groups. Many SES indicators, such as income, employment, and marital status were missing. This study did not measure health and depression. This study was limited to individual-level SES, and future research could also include area-level SES. Despite these limitations, we believe this study still makes a contribution to the literature.

## 5. Conclusions

Our results suggested that in the United States, high EA is associated with a reduced level of perceived stress at work; however, the magnitude of this association depends on the racial and ethnic background of the employee. While highly EA Whites work at low-stress jobs, highly educated AAs and Hispanics experience higher levels of workplace stress. As a result, AAs and Hispanics gain less health benefits from their high EA and employment status.

## Figures and Tables

**Table 1:** Descriptive statistics.

	*n*	%
Race		
White	13,322	84.7
African American (AA)	2398	15.3
Ethnicity		
Non-Hispanic	13,741	87.6
Hispanic	1951	12.4
Gender		
Female	7955	50.6
Male	7765	49.4
Region		
Northeast	2584	16.4
Midwest	3761	23.9
South	5600	35.6
West	3781	24.1
Marital Status		
Other	8429	53.6
Married	7291	46.4
Working at More than One Job		
No	14,271	90.9
Yes	1431	9.1
	***Mean***	***SD***
Age (Years)	43.44	14.20
Educational Attainment (EA)	15.81	2.78
Income	6.86	2.92
Occupational Stress	1.80	0.45

**Table 2:** Correlation Matrix.

	1	2	3	4	5	6	7	8	9	10	11	12	13
1 Race (AAs)	1	−0.13 **	−0.03 **	−0.07 **	−0.01	−0.02 *	−0.07 **	0.21 **	−0.15 **	0.01	−0.06 **	−0.10 **	0.19 **
2 Ethnicity (Hispanics)		1	−0.10 **	0.03 **	0.00	−0.04 **	−0.12 **	0.07 **	−0.01	−0.04 **	−0.28 **	−0.14 **	0.05 **
3 Age			1	0.00	−0.06 **	0.06 **	−0.02 *	−0.03 **	0.18 **	−0.02 **	0.04 **	0.19 **	−0.09 **
4 Gender Male				1	−0.01	−0.01	0.01	−0.03 **	0.07 **	−0.02 *	−0.07 **	0.19 **	0.04 **
5 LGB					1	0.01	−0.02 *	0.01	−0.11 **	0.01	0.04 **	−0.01	0.00
6 Region—Northeast						1	−0.25 **	−0.33 **	0.01	0.01	0.05 **	0.05 **	−0.01
7 Region—Midwest							1	−0.42 **	0.00	0.03 **	0.01	−0.03 **	0.00
8 Region—South								1	−0.02 **	−0.04 **	−0.04 **	−0.04 **	0.01
9 Married									1	−0.04 **	0.09 **	0.20 **	−0.08 **
10 Has One Job										1	0.07 **	−0.01	0.02 **
11 Education Attainment (EA)											1	0.37 **	−0.23 **
12 Income												1	−0.16 **
13 Occupational Stress													1

Notes: Source the National Health Interview Survey (NHIS 2015); CI: confidence interval; SE: standard error; LGB: Lesbian, gay, and bisexual; AA: African American; ** *p* < 0.05. ** *p* < 0.01.

**Table 3:** Two linear regressions with occupational stress as the outcome.

	Standardized Coefficients	B	SE	95% CI		*p*
**Model 1 (Main Effects)**						
Race (AAs)	0.19	0.24	0.01	0.21	0.26	<0.000
Ethnicity (Hispanics)	0.00	0.00	0.01	−0.02	0.03	0.754
Age	−0.06	0.00	0.00	0.00	0.00	<0.000
Gender Male	0.05	0.05	0.01	0.03	0.06	<0.000
LGB	0.01	0.02	0.02	−0.02	0.06	0.268
Region						
Northeast	0.00	0.00	0.01	−0.02	0.03	0.915
Midwest	0.00	0.00	0.01	−0.02	0.02	0.806
South	−0.03	−0.03	0.01	−0.05	−0.01	0.004
Married	−0.01	−0.01	0.01	−0.03	0.01	0.203
Has One Job	0.03	0.05	0.01	0.02	0.07	<0.000
Education Attainment (EA)	−0.19	−0.03	0.00	−0.03	−0.03	<0.000
Income	−0.07	−0.01	0.00	−0.01	−0.01	<0.000
Constant		2.41	0.03	2.36	2.47	<0.000
**Model 2 (M1 + Interactions)**						
Race (AA)	0.03	0.03	0.07	−0.10	0.16	0.627
Ethnicity (Hispanic)	−0.13	−0.18	0.05	−0.28	−0.09	<0.000
Age	−0.06	0.00	0.00	0.00	0.00	<0.000
Gender Male	0.05	0.05	0.01	0.03	0.06	<0.000
LGB	0.01	0.02	0.02	−0.02	0.06	0.256
Region						
Yees						
Northeast	0.00	0.00	0.01	−0.02	0.03	0.893
Midwest	0.00	0.00	0.01	−0.02	0.02	0.920
South	−0.03	−0.03	0.01	−0.05	−0.01	0.005
Married	−0.01	−0.01	0.01	−0.02	0.01	0.263
Has One Job	0.03	0.05	0.01	0.02	0.07	<0.000
Education Attainment (EA)	−0.23	−0.04	0.00	−0.04	−0.03	<0.000
Income	−0.06	−0.01	0.00	−0.01	−0.01	<0.000
Race (AAs) × EA	0.16	0.01	0.00	0.00	0.02	0.002
Ethnicity (Hispanics) × EA	0.13	0.01	0.00	0.01	0.02	<0.000
Constant		2.50	0.03	2.43	2.57	<0.000

Notes: Source the National Health Interview Survey (NHIS 2015); CI: confidence interval; SE: standard error; LGB: Lesbian, gay, and bisexual; AA: African American.
